# Impact of Sleep Quality on Cognitive Function in Elderly Patients With Alzheimer’s Disease

**DOI:** 10.62641/aep.v54i2.2126

**Published:** 2026-04-15

**Authors:** Wanzheng Qi, Jingtong Wang, Xiang Gao, Yanan Liu

**Affiliations:** ^1^Geriatrics Department II, Shijiazhuang People’s Hospital, 050000 Shijiazhuang, Hebei, China; ^2^Department of Geriatric Medicine, Peking University People’s Hospital, 100044 Beijing, China; ^3^Department of Pathogen Biology, Institute of basic Medicine, Hebei Medical University, 050017 Shijiazhuang, Hebei, China; ^4^Key Laboratory of Immune Mechanism and Intervention on Serious Disease in Hebei, Hebei Medical University, 050017 Shijiazhuang, Hebei, China; ^5^Department of Sleep Medicine, Peking University People’s Hospital, 100044 Beijing, China

**Keywords:** Alzheimer’s disease, sleep quality, cognitive dysfunction, polysomnography, sleep architecture

## Abstract

**Objective:**

This study aimed to analyse the effect of sleep quality on cognitive function in elderly patients with Alzheimer’s disease (AD).

**Methods:**

This retrospective study extracted clinical data from the hospital’s electronic medical record system for elderly patients with AD admitted to the Departments of Neurology or Geriatrics between June 2022 and June 2024. Cognitive function was assessed using the Mini-Mental State Examination (MMSE), subjective sleep quality was evaluated with the Athens Insomnia Scale (AIS) and objective sleep architecture parameters were measured via overnight polysomnography (PSG). Participants were stratified into mild and moderate-to-severe cognitive impairment groups according to their MMSE scores. General characteristics and sleep-related indicators were compared between the two groups. A binary logistic regression model was employed to analyse independent factors influencing cognitive impairment severity. In this model, cognitive impairment severity served as the dependent variable, and PSG parameters and AIS score served as the core independent variables. Adjustments were made for potential confounding factors, including age, gender, years of education, disease duration, Hospital Anxiety and Depression Scale (HADS) scores and ‌Instrumental Activities of Daily Living Scale (IADL) scores.

**Results:**

The cohort comprised 61 (40.67%) moderate-to-severe and 89 (59.33%) mild impairment patients. Compared with the mild impairment group, the moderate-to-severe group showed significantly poorer subjective (higher AIS) and objective sleep profiles, including reduced total sleep time, efficiency, and N2/N3 sleep and increased N1 sleep, latency and awakenings (*p* < 0.05). Adjusted regression identified the N3 stage/total sleep time ratio as a protective factor (odds ratio [OR] = 0.720, 95% CI: 0.576–0.900, *p* = 0.004) and the AIS score (OR = 1.850, 95% CI: 1.405–2.434, *p *< 0.001) and number of awakenings (OR = 3.101, 95% CI: 1.879–5.116,* p <* 0.001) as independent risk factors.

**Conclusion:**

In elderly patients with AD, impaired objective sleep architecture and subjective insomnia are significantly associated with poor cognitive function. This study highlighted sleep parameters as potential indicators for cognitive status assessment.

## Introduction

Alzheimer’s disease (AD), the most prevalent cause of dementia in older adults, is clinically characterised by progressive cognitive decline and imposes a substantial burden on patients, families and society [1]. Cognitive impairment severity is influenced by multiple factors. Beyond nonmodifiable risks such as age and genetics, lifestyle factors and comorbid conditions have garnered increasing attention [2]. In recent years, the relationship between sleep quality and cognitive function has become a major research focus. Sleep, a crucial physiological process for maintaining brain health, is commonly impaired in the elderly, manifesting as difficulties in sleep initiation, shallow sleep and early morning awakening [3]. Furthermore, age-related alterations in sleep architecture, specifically an increased proportion of rapid eye movement sleep and a reduction in slow-wave sleep, are considered significant contributors to cognitive decline [4]. Substantial evidence indicates that cognitive impairment and sleep disturbances demonstrate a bidirectional relationship in the aging population, frequently manifesting concurrently during the prodromal stages of dementia [5, 6]. Nevertheless, the precise pathophysiological mechanisms underlying the association between sleep quality and cognitive performance remain incompletely elucidated. Furthermore, dedicated clinical studies examining this relationship within the specific context of elderly patients with AD are yet limited, necessitating further investigation. This research gap prompted the current study to systematically evaluate the impact of sleep quality on cognitive function in this vulnerable population.

## Materials and Methods

### Study Population

This retrospective study extracted clinical data from the hospital’s electronic medical record system for elderly patients with AD admitted to the Departments of Neurology or Geriatrics between June 2022 and June 2024. The inclusion were as follows: ① AD diagnosis conforming to standardised criteria [7], ② informed consent obtained from legally authorised representatives, ③ preserved cognitive and emotional capacity to validly complete all assessments and ④ comprehensive clinical and neuroimaging documentation. The exclusion were as follows: ① significant visual/hearing impairment or aphasia; ② concurrent major organ dysfunction; ③ ongoing participation in alternative clinical studies or premature discontinuation; ④ presence of other systemic disorders known to cause cognitive impairment, such as vascular dementia, dementia with Lewy bodies or Parkinson’s disease dementia; and ⑤ cooccurring primary psychiatric diagnoses. This study was approved by the Ethics Committee of the Shijiazhuang People’s Hospital, China (approval no.: SJZSRMYY-2025-03-02).

### Methods

#### Assessment of Cognitive Impairment

Cognitive evaluation was performed using the Mini-Mental State Examination (MMSE), a validated instrument demonstrating internal consistency of 0.855 as measured by Cronbach’s α [8]. This comprehensive tool assesses multiple cognitive domains including memory retention, temporal and spatial orientation, attentional capacity with computational elements and linguistic capabilities, generating a composite score ranging from 0 to 30. In this study, the MMSE scores were categorised using previously established criteria: scores of 24–30 were defined as within the normal cognitive range, 18–23 as mild cognitive impairment, 10–17 as moderate cognitive impairment and below 10 as severe cognitive impairment [9]. All assessments were administered and interpreted by trained clinical staff following standardised protocols.

#### Assessment of Sleep Quality

Subjective sleep quality over the preceding month was evaluated using the Athens Insomnia Scale (AIS) [10], which demonstrates a Cronbach’s α coefficient of 0.900. This scale comprises eight items assessing sleep induction, nocturnal awakenings, final awakening, total sleep duration, overall sleep quality, daytime well-being, daytime functioning and sleepiness during the day. The total score ranges from 0 to 24. High scores indicate severe sleep disturbances. In this study, a score of ≥6 was established as the positive criterion for insomnia screening [11].

Objective sleep architecture was assessed via overnight polysomnography (PSG) using a Grael PSG system (Australia). All participants underwent at least one full-night (≥8 hours) PSG recording in a standardised hospital room environment. The measured parameters included total sleep time, sleep efficiency, sleep latency, N1 stage duration, N1 stage/total sleep time ratio, N2 stage duration, N2 stage/total sleep time ratio, N3 stage duration, N3 stage/total sleep time ratio and number of awakenings. Sleep stages were defined in accordance with standard nomenclature: N1, N2 and N3 represent the three successive stages of nonrapid eye movement sleep, with N1 marking the transition from wakefulness to sleep, N2 representing established light sleep and N3 corresponding to deep slow-wave sleep. All PSG recordings were conducted by technicians blinded to the participants’ MMSE group allocation. Subsequently, the records were manually scored by a separate technician following the standard criteria established by the American Academy of Sleep Medicine.

### Collection of Baseline Data

Sociodemographic and clinical characteristics were obtained through a review of the electronic medical record system. The collected data included gender, place of residence (rural, urban), body mass index (BMI; dichotomised at ≥24 kg/m^2^), living situation (living alone, living with family), disease duration and marital status (with spouse, without spouse). The following potential confounding factors affecting sleep and cognition were assessed: ① Emotional Status: Anxiety and depressive symptoms were evaluated using the Hospital Anxiety and Depression Scale (HADS) which consists of two 7-item subscales for anxiety (HADS-A) and depression (HADS-D) [12]. Total scores for each subscale range from 0 to 21, with high scores indicating great symptom severity. ② Functional Capacity: The ability to live independently in the community was measured using the Instrumental Activities of Daily Living (IADL) scale [13]. This instrument assesses eight key domains (telephone use, shopping, food preparation, housekeeping, laundry, mode of transportation, responsibility for own medications and ability to handle finances) using a binary scoring system (0 or 1 point per item). The total score ranges from 0 to 8, with low scores denoting great functional dependence and poorer daily living skills. ③ Educational Level: classified as high school and above or below high school.

### Statistical Analysis

Statistical analyses were performed using SPSS software (version 23.0, IBM Corp.; Amonk, NY, USA). The normality of continuous variables was assessed with one-sample Kolmogorov–Smirnov test. Normally distributed data were presented as the mean ± standard deviation ($\bar{x}$ ± S) and compared between groups using independent samples *t*-test. Nonnormally distributed continuous variables were expressed as the median with interquartile range (M [IQR]), and group comparisons were made using Mann–Whitney U test. Categorical data were expressed as numbers and percentages (n [%]) and analysed using chi-square test or Fisher’s exact test when expected cell counts were less than 5. For the construction of a regression model, all variables showing an association with MMSE scores in univariate analysis (*p* < 0.1) and covariates deemed clinically relevant (e.g., age, gender and years of education) were initially entered into the primary model. Variable selection was then performed using a stepwise regression procedure. Multicollinearity was assessed using the variance inflation factor (VIF <5). Logistic regression was employed to analyse the risk factors influencing MMSE scores, with statistical significance set at *p* < 0.05.

## Results

### Cognitive Function in Elderly Patients With AD

The study cohort comprised 150 elderly patients with AD, stratified by cognitive severity into mild (n = 89, 59.33%) and moderate-to-severe (n = 61, 40.67%) groups.

### Comparison of Baseline Characteristics Between the Two Groups

No statistically significant differences regarding gender, place of residence, BMI, living situation, HADS-A score, HADS-D score, educational level, physical function, disease duration, marital status or age were observed between the two groups (*p* > 0.05), suggesting that their baseline characteristics were largely comparable. As expected from the grouping criterion, the MMSE score was significantly lower in the moderate-to-severe cognitive impairment group than in the mild impairment group (*p* < 0.05; Table 1).

**Table 1.** **Comparison of baseline characteristics between the two groups**.

Factor		Moderate-to-severe group (n = 61)	Mild group (n = 89)	Statistical value (χ^2^/*t*)	*p*
Gender	Male	36 (59.02)	53 (59.55)	0.004	0.947
	Female	25 (40.98)	36 (40.45)		
Place of residence	Urban	46 (75.41)	61 (68.54)	0.836	0.361
	Rural	15 (24.59)	28 (31.46)		
Educational level	High School and Above	23 (37.70)	35 (39.33)	0.040	0.841
	Below High School	38 (62.30)	54 (60.67)		
Living situation	Living Alone	9 (14.75)	14 (15.73)	0.027	0.870
	Living with Family	52 (85.25)	75 (84.27)		
Body mass index	≥24 kg/m^2^	39 (63.93)	50 (56.18)	0.902	0.342
	<24 kg/m^2^	22 (36.07)	39 (43.82)		
Marital status	With Spouse	41 (67.21)	67 (75.28)	1.169	0.280
	Without Spouse	20 (32.79)	22 (24.72)		
IADL score (points)		5.55 ± 0.52	5.67 ± 0.55	1.342	0.182
HADS-D score (points)		11.25 ± 1.24	11.00 ± 1.57	1.041	0.300
HADS-A score (points)		10.24 ± 1.18	9.99 ± 1.10	1.327	0.186
MMSE score (points)		17.25 ± 1.52	23.55 ± 1.89	21.665	<0.001
Disease duration	≥5 years	20 (32.79)	35 (39.33)	0.666	0.414
	<5 years	41 (67.21)	54 (60.67)		
Age (years)		70.14 ± 6.54	71.99 ± 5.99	1.790	0.076

Note: IADL, Instrumental Activities of Daily Living scale; HADS-D, Hospital Anxiety and Depression Scale-Depression; HADS-A, Hospital Anxiety and Depression Scale-Anxiety; MMSE, Mini-Mental State Examination.

### Comparison of Subjective and Objective Sleep-Related Indices Between the Two Groups

Compared with the mild impairment group, the moderate-to-severe cognitive impairment group exhibited a more impaired sleep profile, characterised by higher subjective AIS scores. Objectively, PSG recordings revealed shorter total sleep time, lower sleep efficiency, longer sleep latency and greater number of awakenings per hour in the moderate-to-severe group. Regarding sleep architecture, the absolute duration and percentage of total sleep time for N2 and N3 sleep stages were significantly reduced in the moderate-to-severe group. By contrast, the percentage of N1 sleep was significantly elevated (*p* < 0.05; Table 2).

**Table 2.** **Comparison of AIS scores and polysomnographic parameters between the two groups**.

Factor	Moderate-to-severe group (n = 61)	Mild group (n = 89)	*t*	*p*
AIS score (points)	11.25 ± 1.56	4.12 ± 1.38	29.468	<0.001
Total sleep time (min)	335.15 ± 25.28	400.36 ± 30.50	13.766	<0.001
Sleep efficiency (%)	65.15 ± 7.28	86.34 ± 8.98	15.299	<0.001
Number of awakenings (times/h)	7.15 ± 0.54	4.58 ± 0.35	35.373	<0.001
Sleep latency (min)	31.15 ± 9.18	16.37 ± 6.52	11.533	<0.001
N1 duration (min)	15.15 ± 3.25	10.15 ± 4.48	7.470	<0.001
N1 stage/Total sleep time (%)	4.52 ± 0.24	2.54 ± 0.18	57.702	<0.001
N2 duration (min)	190.12 ± 35.24	240.35 ± 40.58	7.848	<0.001
N2 stage/Total sleep time (%)	56.73 ± 4.24	60.03 ± 5.48	3.959	<0.001
N3 duration (min)	55.10 ± 4.20	85.35 ± 8.50	25.708	<0.001
N3 stage/Total sleep time (%)	16.44 ± 2.21	21.32 ± 2.79	11.421	<0.001

Note: AIS, Athens Insomnia Scale.

### Binary Logistic Regression Analysis of Factors Influencing the Severity of Cognitive Impairment

With cognitive impairment severity (0 = mild, 1 = moderate-to-severe) as the dependent variable, the variables significant in the univariate analysis and the clinically important covariates were entered into a binary logistic regression model. Following stepwise selection, the variables retained in the final model and their corresponding results are presented in Table 3. After adjustments were made for age, gender, years of education, disease duration, IADL scores and HADS scores, the N3 stage/total sleep time ratio was identified as a protective factor (odds ratio [OR] = 0.720, 95% CI: 0.576–0.900, *p* = 0.004). This finding indicates that for each unit increase in the proportion of N3 sleep, the risk of belonging to the moderate-to-severe cognitive impairment group was reduced by approximately 28%. Conversely, the AIS total score (OR = 1.850, 95% CI: 1.405–2.434, *p* < 0.001) and number of awakenings (OR = 3.101, 95% CI: 1.879–5.116, *p* < 0.001) were independent risk factors. This result suggests that severe subjective insomnia symptoms and frequent nocturnal awakenings were significantly associated with a poor cognitive impairment status.

**Table 3.** **Binary logistic regression analysis of factors influencing the severity of cognitive impairment**.

Variable	β	S.E.	Wald	OR	*p*	95% CI
Constant	–8.549	2.101	16.556	-	<0.001	-
N3 stage/Total sleep time	–0.339	0.114	8.298	0.720	0.004	0.576–0.900
AIS score	0.615	0.141	19.012	1.850	<0.001	1.405–2.434
Number of awakenings	1.131	0.255	19.698	3.101	<0.001	1.879–5.116
Age	0.045	0.028	2.546	0.111	1.046	0.989–1.106

Note: AIS, Athens Insomnia Scale; S.E., standard error; OR, odds ratio.

## Discussion

Sleep represents an evolutionarily conserved biological process that undergoes substantial transformations in qualitative and structural dimensions with advancing age. Jean-Louis *et al*. [14] further substantiated that aging drives fundamental biological modifications in sleep regulation mechanisms, accounting for the elevated prevalence of sleep–wake disturbances observed in older populations. Although investigations of sleep disorders in the elderly yielded heterogeneous methodological approaches, they collectively established a consistent association between impaired sleep quality and cognitive deterioration and suggested that suboptimal sleep constitutes a modifiable risk factor for cognitive decline and dementia progression [15, 16]. Within this framework, elderly patients with AD represent a distinctive neurodegenerative context characterised by established cognitive deficits. The critical question of whether sleep disturbances potentiate existing cognitive impairment in this vulnerable cohort require a systematic investigation.

Potvin *et al*. [17] suggested that poor sleep quality serves as an early indicator of cognitive decline. The findings of the present study revealed that compared with the mild cognitive impairment group, the moderate-to-severe group exhibited a more compromised sleep profile, characterised by higher subjective AIS scores and objective PSG measures indicating shorter total sleep time, lower sleep efficiency, longer sleep latency and greater number of awakenings. Regarding sleep architecture, the absolute duration and percentage of total sleep time for N2 and N3 stages were significantly reduced in the moderate-to-severe group, whereas the percentage of N1 sleep was markedly elevated. Binary logistic regression analysis demonstrated that after the adjustment for confounding factors, the N3 stage/total sleep time ratio served as a protective factor, whilst the number of awakenings constituted an independent risk factor. This result suggests that poor objective sleep architecture, particularly reduced deep sleep and increased sleep fragmentation, and severe subjective insomnia symptoms are significantly associated with poor cognitive performance, aligning with the aforementioned research. Furthermore, our results clarify that a reduction in N3 sleep is associated with poor immediate cognitive status, consistent with the theory of sleep’s neurorestorative function. Different from previous works focusing on populations with mild cognitive impairment [18], the present study did not find sleep latency or total sleep time to be independently associated with cognitive scores after adjusting for other factors. This finding may suggest that in the advanced stage of AD, the depth and continuity of sleep become more critical than mere sleep duration. Given the retrospective design of the current study, these findings only reveal a statistical association between sleep parameters and cognitive function and cannot establish temporal sequence or causality.

The underlying mechanisms are hypothesised to involve the following pathways. Firstly, β-amyloid (Aβ) metabolism is critically influenced by sleep–wake cycles. The brain’s glymphatic system, most active during deep sleep stages, facilitates the clearance of neurotoxic metabolites including Aβ peptides, with clearance rates approximately doubling during sleep relative to those whilst awake [19]. Chronic sleep disruption compromises this clearance mechanism, leading to accelerated Aβ accumulation that initiates synaptic dysfunction and neuronal damage through multiple pathways. Emerging evidence indicates that cerebral Aβ deposition reciprocally impairs nonrapid eye movement sleep stability and reduces slow-wave activity, establishing a vicious cycle between deteriorating sleep quality and progressive neurodegeneration in memory-related circuits [20]. Thus, the observed reduction in N3 sleep in this study may represent a consequence of disrupted sleep architecture due to accumulating Aβ pathology in the brain and a contributing factor that accelerates pathological progression and cognitive decline through diminished clearance efficiency. Secondly, chronic sleep fragmentation (elevated number of awakenings) and subjective insomnia (high AIS scores) are often accompanied with the activation of the hypothalamic–pituitary–adrenal axis and sympathetic nervous system. This phenomenon leads to increased levels of pro-inflammatory glucocorticoids and catecholamines, which promote the release of inflammatory cytokines from immune cells and trigger chronic neuroinflammation. Such persistent neuroinflammation can inflict damage upon critical brain regions such as the hippocampus and represents a core mechanism underlying AD progression [21, 22]. Consequently, the association between sleep fragmentation and poor cognitive performance observed in this study may be partially mediated by this shared pathway of neuroinflammation. Thirdly, circadian rhythm disruption affects melatonin secretion patterns. The progressive deterioration of sleep architecture in patients with AD disrupts the normal nocturnal surge of melatonin, a hormone possessing sleep-regulating properties and neuroprotective effects through antioxidant, anti-inflammatory and neurotrophic mechanisms [23]. Diminished melatonin levels consequently exacerbate sleep disturbances whilst reducing endogenous protection against oxidative stress and inflammation, further compromising hippocampal integrity and synaptic plasticity [24]. Thus, AD pathology may impair sleep-regulating centres, compromising objective sleep architecture (particularly reduced deep sleep and increased fragmentation) and exacerbating subjective insomnia symptoms. Conversely, sleep deterioration may further engage in a bidirectional vicious cycle with cognitive dysfunction through mechanisms such as diminished cerebral Aβ clearance, aggravated chronic neuroinflammation and disrupted melatonin secretion rhythms.

The conclusions drawn from this study represent plausible inferences based on the literature. The causal relationship and temporal sequence between sleep disturbances and cognitive impairment require confirmation through prospective cohort studies and interventional trials (e.g., sleep-targeted interventions). Although this study utilised subjective and objective sleep assessment tools and comprehensively adjusted for demographic, emotional and functional confounders, several limitations should be acknowledged. Firstly, the single-centre sample may be subjected to selection bias. Secondly, the single-night PSG recording may have been influenced by the first-night effect. Thirdly, despite controlling for multiple covariates, residual confounding from unmeasured factors, such as APOE ε4 genotype and concomitant medications, cannot be ruled out. Future research should aim to clarify whether sleep disturbances can predict the trajectory of cognitive decline in patients with AD and explore the potential therapeutic value of improving sleep (e.g., enhancing slow-wave sleep) for mitigating cognitive deterioration.

## Conclusion

In elderly patients with AD, impairments in objective sleep architecture—particularly reduced deep sleep and increased sleep fragmentation—and severe subjective insomnia complaints are independently and significantly associated with poor cognitive performance scores.

## Availability of Data and Materials

The datasets used and/or analyzed in this study are available from the corresponding author according to a reasonable request.
